# Supplementary material for: Outcomes of Radiotherapy for Mesenchymal and Non-Mesenchymal Subtypes of Gastric Cancer
Source: Cancers (Basel). 2020 Apr 10;12(4):943. doi: 10.3390/cancers12040943 (PMC7226608; doi:10.3390/cancers12040943)
Supplement: Supplementary file 1 [file cancers-12-00943-s001.pdf]

Supplementary Materials

Table S1. Baseline characteristics of enrolled patients of present study and all patients of ARTIST trial.

**Variables**		**ARTIST (n = 458, %)**	**Present study (n = 106, %)**	**p**
Age – yr	Median	56	54	0.21
	Range	28-76	35-76	
Sex	Male	296 (64.6)	67 (63.2)	0.43
	Female	162 (35.4)	39 (36.8)	
Macroscopic type	0 (superficial)	53 (11.6)	4 (3.8)	<0.001
	1 (mass)	8 (1.7)	1 (0.9)	
	2 (ulcerative)	91 (19.9)	21 (19.8)	
	3 (ulceroinfiltrative)	219 (47.8)	57 (53.8)	
	4 (diffuse infiltrative)	45 (9.8)	23 (21.7)	
	5 (unclassifiable)	42 (9.2)	0 (0.0)	
Type of operation	Total	161 (35.2)	48 (45.3)	0.04
	Subtotal	296 (64.8)	58 (54.7)	
Lauren classification	Diffuse	74 (69.8)	274 (59.8)	0.10
	Intestinal	27 (25.5)	163 (35.6)	
	Unclassified	5 (4.7)	21 (4.6)	
No of dissected LNs	Median	40	42	0.29
	Range	12-142	14-96	
No of positive LNs	Median	9	3	<0.001
	Range	0-50	0-51	
AJCC 8th stage	I	0 (0.0)	28 (6.1)	<0.001
	II	2 (1.8)	187 (40.8)	
	IIIA	56 (52.8)	115 (25.1)	
	IIIB	22 (20.8)	86 (18.8)	
	IIIC	26 (24.5)	42 (9.2)	
LVI	Positive	84 (79.2)	292 (63.8)	0.004
	Negative	21 (19.8)	155 (33.8)	
	Unknown	1 (0.9)	11 (2.4)	
PNI	Positive	63 (59.4)	207 (45.2)	0.01
	Negative	40 (37.7)	234 (51.1)	
	Unknown	3 (2.8)	17 (3.7)	

Abbreviations: LN = lymph node, AJCC = American Joint Committee on Cancer, LVI = lymphovascular invasion, PNI = perineural invasion

Table S2. Baseline characteristics of patients according to adjuvant treatment.

**Variables**		**XP-RT (n = 56, %)**	**XP (n = 50, %)**	**p**
Age – yr	Median	49	57	0.04
	Range	35-76	38-75	
Sex	Male	32 (58.2)	34 (68.0)	0.20
	Female	23 (41.8)	16 (32.0)	
Macroscopic type	0 (superficial)	3 (5.5)	1 (2.0)	0.84
	1 (mass)	0 (0.0)	0 (0.0)	
	2 (ulcerative)	1 (1.8)	0 (0.0)	
	3 (ulceroinfiltrative)	10 (18.2)	10 (20.0)	
	4 (diffuse infiltrative)	28 (50.9)	29 (58.0)	
Type of operation	Total	28 (50.0)	20 (40.0)	0.33
	Subtotal	28 (50.0)	30 (60.0)	
Lauren classification	Diffuse	37 (66.1)	37 (74.0)	0.44
	Intestinal	17 (30.4)	10 (20.0)	
	Unclassified	2 (3.6)	3 (6.0)	
No of dissected LNs	Median	40	43	0.66
	Range	18-75	14-96	
No of positive LNs	Median	8	8	0.91
	Range	0-50	1-24	
AJCC 8th stage	II	1 (1.8)	1 (0.0)	0.63
	IIIA	30 (53.6)	26 (26.0)	
	IIIB	11 (19.6)	11 (52.0)	
	IIIC	14 (20.0)	12 (22.0)	
LVI	Positive	46 (82.1)	38 (76.0)	0.54
	Negative	10 (17.9)	11 (22.0)	
	Unknown	0 (0.0)	1 (2.0)	
PNI	Positive	34 (60.7)	29 (58.0)	0.82
	Negative	21 (37.5)	19 (38.0)	
	Unknown	1 (1.8)	2 (4.0)	
Molecular subtype	Mesenchymal	19 (33.9)	17 (34.0)	1.00
	Non-mesenchymal	37 (66.1)	33 (66.0)	

Abbreviations: XP: capecitabine and cisplatin, XP-RT: XP-radiation therapy, LN: lymph node, AJCC: American Joint Committee on Cancer, LVI: lymphovascular invasion, PNI: perineural invasion

Table S3. Detailed patterns of recurrence according to subtype and adjuvant treatment.

	Mesenchymal			Non-mesenchymal		
	**XP-RT (n = 19)**	**XP (n = 17)**	**p**	**XP-RT (n = 37)**	**XP (n = 33)**	**p**
Loco-regional recurrence	2 (10.5)	5 (29.4)	0.219	6 (16.2)	7 (21.2)	0.760
Distant lymph node	0 (0.0)	1 (5.9)	0.472	0 (0.0)	4 (12.1)	0.045
Peritoneal seeding	9 (47.4)	7 (41.2)	0.749	9 (24.3)	7 (21.2)	0.784
Hematogenous metastasis	0 (0.0)	1 (5.9)	0.472	3 (8.1)	3 (9.1)	1.000
Total distant metastasis	9 (47.4)	9 (52.9)	1.00	12 (32.4)	11 (33.3)	1.000

Abbreviations: XP: capecitabine and cisplatin, XP-RT: XP-radiation therapy

Table S4. Multivariate analysis of recurrence-free survival (RFS), and overall survival (OS) in mesenchymal and non-mesenchymal subtypes including perineural invasion.

			RFS			OS		
**variable**	**Factor**	**Reference**	**p**	**HR**	**95% CI**	**p**	**HR**	**95% CI**
**Mesenchymal subtype**								
Stage	IIIB	II/IIIA	**0.015**	6.19	1.43-26.76	0.246	2.19	0.58-8.23
	IIIC		0.107	2.64	0.81-8.52	0.065	2.70	0.94-7.75
Type of operation	TG	STG	**0.033**	3.24	1.10-9.59	0.340	1.56	0.63-3.87
PNI	positive	negative	0.538	1.45	0.44-4.77	0.242	1.89	0.65-5.52
Lauren type	other	diffuse	0.309	0.28	0.03-3.22	0.566	1.63	0.31-8.68
ARTIST	XP-RT	XP	0.916	0.94	0.31-2.89	0.061	2.77	0.95-8.01
**Non-mesenchymal subtype**								
Stage	IIIB	II/IIIA	0.284	1.73	0.64-4.69	0.241	1.82	0.67-4.94
	IIIC		**<0.001**	6.53	2.32-18.39	**<0.001**	7.74	2.63-22.77
Type of operation	TG	STG	0.093	1.94	0.90-4.19	**0.009**	2.88	1.31-6.36
PNI	positive	negative	**0.044**	2.71	1.03-7.14	0.109	2.14	0.84-5.45
Lauren type	other	diffuse	**0.017**	0.31	0.12-0.81	**0.018**	0.30	0.11-0.81
ARTIST	XP-RT	XP	0.403	0.69	0.29-1.64	0.250	0.59	0.24-1.45

Abbreviations: RFS: recurrence-free survival, OS: overall survival, HR: hazard ratio, CI: confidence interval, TG: total gastrectomy, STG: subtotal gastrectomy, XP: capecitabine and cisplatin, XP-RT: XP-radiation therapy
